# Comparison of Efficacy of Topical Betamethasone Dipropionate and Topical Minoxidil in Patients With Alopecia Areata

**DOI:** 10.7759/cureus.56282

**Published:** 2024-03-16

**Authors:** Sana Aslam, Aqsa Z Awan, Madiha M Iqbal, Saira Saeed, Mariyam Saeed, Zartaj Liaqat, Saad Abdullah Dar, Salamat Ali, Muhammad Ahsan Asif, Haseeb Mehmood Qadri

**Affiliations:** 1 Dermatology, Jinnah Hospital, Lahore, PAK; 2 Medicine, University Hospital Birmingham, Birmingham, GBR; 3 Medicine, Jinnah Hospital, Lahore, PAK; 4 Medicine, Aziz Bhatti Shaheed Teaching Hospital, Gujrat, PAK; 5 Surgery, Lahore General Hospital, Lahore, PAK

**Keywords:** alopecia, pakistan, puva, ultraviolet therapy, psoralen, efficacy, minoxidil, betamethasone, topical, alopecia areata

## Abstract

Background and objective

Alopecia areata (AA) is a reiterative and nonscarring type of hair loss that can affect any hairy area of the body, particularly the scalp. It manifests as patchy or confluent hair loss with variations in demographics and ethnicity. There are numerous treatment options available, including topical and systemic steroids, topical minoxidil, dithranol, tacrolimus, psoralen and ultraviolet therapy (PUVA), contact immunotherapy, and oral immunosuppressive drugs. However, no previous contrast for efficacy is present between the topical betamethasone versus topical minoxidil alone in our population.

This study aims to compare the efficacy of topical betamethasone dipropionate versus topical minoxidil in patients with AA.

Methodology

A nonrandomized controlled study was conducted at the Department of Dermatology, Jinnah Hospital Lahore, incorporating the data of patients between July 26, 2016, and January 26, 2017, after obtaining institutional ethical approval. One hundred patients with alopecia, either on the scalp or any other hairy part, from both genders, aged between 18 and 50 years, were included in the study. Two groups were created, and patients were assigned to these groups based on the clinician's choice. Group A patients were administered betamethasone dipropionate (0.05%) lotion twice daily on affected areas for 12 weeks. Group B patients were administered minoxidil (5%) solution twice daily on affected areas for 12 weeks. A four-week follow-up plan was followed. A five-point scale score system was used for alopecia grading. After 12 weeks, the hair regrowth score (RGS) was used to compare the efficacy of treatment between the two groups.

Results

A total of 100 patients with grades S1 to S3 AA of less than three months duration were enrolled. Two groups were created, with 50 patients in each group. The mean age in Group A was 29.08 ± 6.51 years, while in Group B, it was 29.38 ± 6.62 years. In Group A, there were 76% males and 24% females, while in Group B, there were 74% males and 26% females. Comparison of efficacy of topical betamethasone dipropionate versus topical minoxidil in patients with AA demonstrated a greater efficacy of 74% (Grade 3 and Grade 4 responses) in Group A, while in Group B, only 42% of patients showed efficacy. A statistically significant difference was found, with a *P*-value of 0.001. No serious side effects were noted.

Conclusions

Our study concluded that topical betamethasone dipropionate (0.05%) lotion has statistically significantly higher efficacy compared to topical minoxidil (5%) solution in patients with AA.

## Introduction

Alopecia areata (AA) is an autoimmune disorder that is embodied by nonscarring hair loss on any area of the body having hairs, particularly the scalp [1]. Clinical presentation contrasts from either the patchy hair loss that may be well-defined or the diffuse participation of the scalp or the body [1]. AA has a prevalence of about 2% in the general population, including all ages, genders, and various ethnic groups [2]. No gender discrimination exists in the prevalence of AA; however, one of the most known autoimmune diseases in men is AA [3]. AA can occur among any age group, but the prevalence is common among children when compared to the adult population [4]. It is an autoimmune disorder with various factors, including psychiatric issues, endocrine abnormalities, genetic problems, and infectious agents, all significant contributors to the condition [5]. The diagnosis is usually made based on clinical manifestations; however, histopathology and trichoscopy are important clinically [1].

There is no conclusive cure for AA, but common treatment modalities include topical, intralesional, and systemic corticosteroids, topical minoxidil, anthralin, topical immune modulators, psoralen and ultraviolet therapy (PUVA), cyclosporine, azathioprine, tacrolimus, sulfasalazine, mesotherapy, and biologic response modifiers [6,7]. Treatment efficacy has been hindered by the spontaneous remission and relapsing nature and rate of the disease process [7]. The use of 5% topical minoxidil has demonstrated better efficacy in contrast to 1% minoxidil in AA treatment [8].

There is a paucity of present English scientific literature comparing the use of topical betamethasone dipropionate versus topical minoxidil for AA. After the literature search from PubMed and Google Scholar, we conclude that our study is the first of its kind from Pakistan comparing these two treatment options for AA. We aimed to compare the efficacy of topical betamethasone dipropionate versus topical minoxidil in patients with AA.

## Materials and methods

This prospective, nonrandomized controlled study was conducted in the Department of Dermatology, Jinnah Hospital, Lahore, for six months between July 26, 2016, and January 26, 2017. Ethical approval for the study was obtained from the Ethical Review Board of the mentioned hospital, with reference number 63/2016-AIMC, on July 1, 2016. The sample size of 100 patients, with 50 in each group was calculated with 80% power of the test, a 5% significance level using the nonprobability sampling technique. Patients with AA of S1 to S3 grade, aged 18 to 49 years irrespective of gender and with disease duration less than three months were included in this study. Patients under 18 years of age, those with a disease duration exceeding three months, pregnant and lactating women, individuals with a positive history of allergy to minoxidil, and patients with scars on the bald patches were excluded from the study.

Patients with AA who met the inclusion criteria were split into two groups through the draw method, with equal patients in each group. For 12 weeks, Group A patients applied betamethasone dipropionate (0.05%) lotion twice daily and Group B patients applied topical minoxidil (5%) solution twice daily. A four-week follow-up period was conducted for patients in both groups to assess compliance. The five-point scale score used for the assessment of alopecia grading is described in Table 1.

**Table 1:** Five-point scale score for alopecia grading.

Score	Percentage of hair loss
S1	<10%
S2	11%-25%
S3	26%-50%
S4	51%-75%
S5	>75%

After 12 weeks, treatment efficacy was evaluated using the hair regrowth score (RGS), with a scale ranging from 0 to 4 (Table 2).

**Table 2:** Hair regrowth score.

Score	Percentage of hair regrowth
0	<10%
1	11%-24%
2	25%-49%
3	50%-75%
4	>75%

Data entry and analysis were done using IBM SPSS Statistics for Windows, Version 22.0 (IBM Corp., Armonk, NY). Measures of central tendency (mean, mode, median, and frequency) were calculated for categorical variables such as age, gender, and HRS. Percentage and frequency were calculated for gender and efficacy of two drugs. Measures of central dispersion (standard deviation [SD], confidence interval, and variance) were assessed for quantitative variables. We used the chi-square test for comparison of efficacy for the two groups. A *P*-value less than or equal to 0.05 was considered significant. 

## Results

One hundred patients were included in the study. Two groups were created, with 50 patients in each group. The mean age ± SD calculated in Group A was 29.08 ± 6.51 years, while in Group B, it was 29.38 ± 6.62 years. The age distribution of the patients showed that 64% in Group A and 56% in Group B were aged between 18 and 30 years, while 36% in Group A and 44 % in Group B were aged between 31 and 50 years (Table 3).

**Table 3:** Age distribution of patients included in both groups, with mean and standard deviation (SD).

Age (in years)	Group A (*n *= 50)		Group B (*n *= 50)	
	Number of patients	%	Number of patients	%
18-30	32	64	28	56
31-50	18	36	22	44
Total	50	100	50	100
Mean ± SD	29.08 ± 6.51		29.38 ± 6.62	

Gender distribution showed that 76% of patients in Group A were males and 24% of patients were females, while 74% of patients in Group B were males and 26% of patients were females (Table 4).

**Table 4:** Gender distribution of patients included in both groups.

Gender	Group A (*n *= 50)		Group B (*n *= 50)	
	Number of patients	%	Number of patients	%
Male	38	76	37	74
Female	12	24	13	26
Total	50	100	50	100

Comparison of efficacy of topical betamethasone dipropionate versus topical minoxidil in patients with AA showed that in Group A, 74% showed efficacy, while in Group B, only 42% showed efficacy, clearly implying better efficacy of topical betamethasone dipropionate over topical minoxidil (Table 5).

**Table 5:** Comparison of efficacy of topical betamethasone dipropionate versus topical minoxidil in patients with alopecia areata (N = 100). *P*-value = 0.0012.

Efficacy	Group A (*n *= 50)		Group B (*n *= 50)	
	Number of patients	%	Number of patients	%
Yes	37	74	21	42
No	13	26	29	58
Total	50	100	50	100

Data were stratified for both age groups and both genders. *P*-values of all parameters were statistically significant, i.e., <0.05, and indicated the efficacy of drugs given in respective groups (Table 6).

**Table 6:** Stratification of efficacy of drugs with respect to age groups and gender groups where given P-values are significant, i.e., P-values < 0.05.

Group characteristics	Efficacy response		*P*-value
	Yes	No	
Group A with age 18-30 years	23	9	0.04
Group B with age 18-30 years	13	15	
Group A with age 31-50 years	14	4	0.008
Group B with age 31-50 years	8	14	
Group A with male gender	27	11	0.01
Group B with male gender	16	21	
Group A with female gender	10	2	0.02
Group B with female gender	5	8	

**Figure 1:**
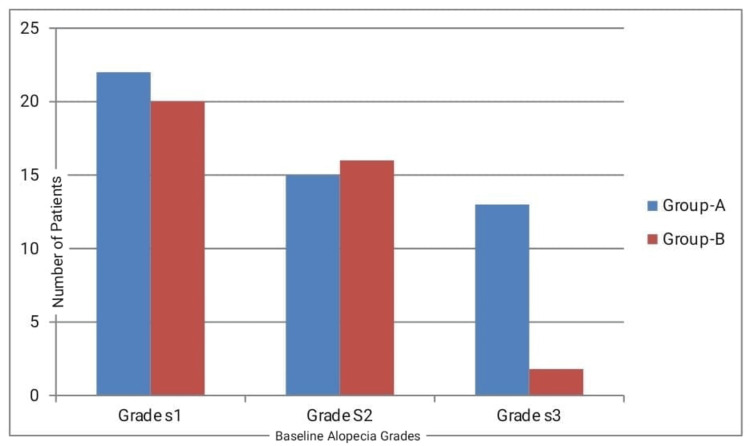
Baseline alopecia grading for both groups.

The severity of the disease was graded according to the five-point score. The distribution of patients according to severity is depicted in Figure 1. In Group A, 44% of patients and in Group B, 40% of patients were classified in the S1 severity grade. The majority of patients in both groups were in the S1 grade of alopecia, indicating less than 10% hair loss. However, there were more patients in the S3 group in Group A compared to Group B.

## Discussion

AA is a recurring disorder characterized by either patchy, nonscarring, or confluent hair loss, affecting any hair-bearing area of the body, especially the scalp [1]. It can manifest as AA universalis, totalis, or ophiasis type [9]. AA is a disorder of autoimmune nature that can also occur in the background of a different autoimmune disorder such as thyroid disease and vitiligo [10]. Several environmental factors are also associated with the pathogenesis of AA, such as psychological stress, infections, toxins, along with autoimmunity [11].

The prevalence of AA is common among the young population and children as compared to adults [4]. In our study, the selected patients fall into the age group of 18-50 years, with the mean ages in Group A 29.08 years and in Group B 29.38 years. No gender discrimination is present in the prevalence of AA; however, it is one of the most common autoimmune disorders in men [3]. This was comparable to our study in which 100 patients were divided into two groups, with each having 50 patients with a male prevalence of 76% in Group A and 74% in Group B.

Several different treatment options are available, such as topical and systemic steroids, topical minoxidil, dithranol, tacrolimus, tretinoin, PUVA, contact immunotherapy, and oral immunosuppressive drugs [6,7]. In our study, we used topical minoxidil and topical betamethasone dipropionate therapy. Minoxidil was originally used to treat high blood pressure, and hair growth was observed as a side effect of the drug [12]. Topical minoxidil solution, especially its 2% and 5% preparations, was found to be efficacious in treating AA [13]. Suchonwanit et al. document an elaborated review of minoxidil's utility in pathologies of hair growth, including androgenic alopecia, chemotherapy-induced alopecia, scarring alopecia, as well as AA. The use of the 5% minoxidil solution has corroborated an astonishing result of 81% in the regrowth of terminal hairs [14]. We used the 5% formulation of minoxidil in our study. However, there was no prior comparison done between topical betamethasone dipropionate alone versus topical minoxidil 5% alone in our population.

Baseline alopecia grading showed that the majority of patients in our study belonged to the S1 grade (<10% hair loss). The 0.05% formulation of topical betamethasone dipropionate has shown promising results in the treatment of AA [15]. The results of our study demonstrate similar findings when comparing the efficacy of hair regrowth in both groups. Specifically, the betamethasone dipropionate group exhibited 74% efficacy, while the minoxidil group showed only 42% efficacy. This difference was statistically significant (*P*-value = 0.001).

Various trials of betamethasone have been conducted to compare its efficacy against weekly pulse azathioprine therapy and oral cyclosporine. These trials have documented weaker responses of betamethasone in the treatment of moderate-to-severe AA. Gupta et al. recently conducted a randomized controlled trial, comparing weekly azathioprine pulse and betamethasone in selected cohorts. Results were considerably effective in both groups [16]. Jang et al. reported a patient satisfaction of 43.20% in their retrospective study of patients with AA [17]. However, the results of our study indicate that topical betamethasone dipropionate (0.05%) applied twice daily for 12 weeks shows a higher efficacy as compared to topical minoxidil (5%) solution in patients with AA.

Limitations

The authors acknowledge the limitations of the study. The small sample size and limited existing literature on the same topic are reasons why constructive comparisons could not be made. There was no placebo or control group to compare the effectiveness of topical betamethasone dipropionate (0.05%) lotion and topical minoxidil (5%) solution. Although topical betamethasone dipropionate (0.05%) lotion is an effective and less expensive treatment option and has higher efficacy than topical minoxidil (5%) solution, long-term efficacy and safety along with effective doses of these drugs were not studied and need further studies to check the relapse of AA after complete cessation of the treatment.

Clinical recommendations

Both betamethasone dipropionate (0.05%) lotion and minoxidil (5%) lotion are effective in the treatment of AA. However, the efficacy and results are better for betamethasone dipropionate (0.05%), so its use should be implemented in clinical settings. Educating dermatologists and clinicians about the superior results of betamethasone dipropionate therapy for the treatment of AA can be beneficial for patients, leading to more effective outcomes.

## Conclusions

As both the topical betamethasone dipropionate (0.05%) lotion and the topical minoxidil 5% solution are aimed at hair regrowth in patients with AA, our study proves twice daily application of topical betamethasone dipropionate (0.05%) lotion therapy for 12 weeks in patients with AA provides higher efficacy results than topical minoxidil that are statistically significant. A better response was observed in the younger population through the use of topical betamethasone dipropionate (0.05%) lotion compared to older patients with the mean age. Although the use of either treatment is safe, earlier presentation and diagnosis of AA and concurrent use of topical betamethasone dipropionate (0.05%) lotion therapy provided higher efficacy and beneficial results than minoxidil therapy.
